# Do aluminium-based phosphate binders continue to have a role in contemporary nephrology practice?

**DOI:** 10.1186/1471-2369-12-20

**Published:** 2011-05-13

**Authors:** David W Mudge, David W Johnson, Carmel M Hawley, Scott B Campbell, Nicole M Isbel, Carolyn L van Eps, James JB Petrie

**Affiliations:** 1 Department of Nephrology, University of Queensland at Princess Alexandra Hospital, Ipswich Road, Woolloongabba, Queensland, Australia

## Abstract

**Background:**

Aluminium-containing phosphate binders have long been used for treatment of hyperphosphatemia in dialysis patients. Their safety became controversial in the early 1980's after reports of aluminium related neurological and bone disease began to appear. Available historical evidence however, suggests that neurological toxicity may have primarily been caused by excessive exposure to aluminium in dialysis fluid, rather than aluminium-containing oral phosphate binders. Limited evidence suggests that aluminium bone disease may also be on the decline in the era of aluminium removal from dialysis fluid, even with continued use of aluminium binders.

**Discussion:**

The K/DOQI and KDIGO guidelines both suggest avoiding aluminium-containing binders. These guidelines will tend to promote the use of the newer, more expensive binders (lanthanum, sevelamer), which have limited evidence for benefit and, like aluminium, limited long-term safety data. Treating hyperphosphatemia in dialysis patients continues to represent a major challenge, and there is a large body of evidence linking serum phosphate concentrations with mortality. Most nephrologists agree that phosphate binders have the potential to meaningfully reduce mortality in dialysis patients. Aluminium is one of the cheapest, most effective and well tolerated of the class, however there are no prospective or randomised trials examining the efficacy and safety of aluminium as a binder. Aluminium continues to be used as a binder in Australia as well as some other countries, despite concern about the potential for toxicity. There are some data from selected case series that aluminium bone disease may be declining in the era of reduced aluminium content in dialysis fluid, due to rigorous water testing.

**Summary:**

This paper seeks to revisit the contemporary evidence for the safety record of aluminium-containing binders in dialysis patients. It puts their use into the context of the newer, more expensive binders and increasing concerns about the risks of calcium binders, which continue to be widely used. The paper seeks to answer whether the continued use of aluminium is justifiable in the absence of prospective data establishing its safety, and we call for prospective trials to be conducted comparing the available binders both in terms of efficacy and safety.

## Background

Oral phosphate binding agents have been used for the treatment of hyperphosphatemia in dialysis patients for decades. Their safety record was marred in the early 1980's by reports of aluminium-related neurological and bone disease, and so-called "dialysis dementia" led to limitations or complete avoidance of their use and a search for alternative agents. Initially the neurological syndrome was attributed to orally administered aluminium, but historical evidence suggests that severe toxicity may have primarily been caused by excessive exposure to aluminium in dialysis fluid, rather than aluminium-containing oral phosphate binders. There were reports of epidemics of dementia in specific dialysis centres were the aluminium content of the water used for dialysis was later found to be very high, suggesting that aluminium loading of patients was occurring from the water. With the advent of stringent testing of dialysis water for aluminium and other minerals and their removal by reverse-osmosis, it became possible to remove aluminium almost completely from dialysis water. Since then "dialysis dementia" has completely disappeared, even in countries where aluminium continues to be used as a binder. A small body of evidence suggests that aluminium bone disease may also be on the decline in the era of aluminium removal from dialysis fluid, even with continued use of aluminium binders. Contemporary guidelines for the treatment of hyperphosphatemia in dialysis patients continue to recommend avoidance or restriction of aluminium binders due to the concerns of potential neurological toxicity and advocate the use of newer, more expensive binders whose long term safety remains unclear. Prospective randomised trials of the different agents for phosphate binding to establish both their safety and efficacy in terms of hard clinical endpoints are lacking, and should be pursued by the nephrology community.

## Discussion

### 1. Historical evidence suggests that water, not binders, was the source of previous cases of aluminium toxicity in dialysis patients

The problem of phosphate accumulation became apparent quite early in the history of dialysis. Soft tissue and vascular calcification could often be clearly visualised on plain x-ray because of calcium phosphate deposition in the vessel walls. As phosphate is present in most foods, dietary restriction of phosphate was not an adequate solution. The treatment of hyperphosphatemia was intensified by the additional mealtime administration of phosphate binders, such as aluminium hydroxide and calcium carbonate. Phosphate accumulation was also identified early as an important cause of secondary hyperparathyroidism. Slatopolsky demonstrated in dogs with surgically induced renal failure that hyperparathyroidism was universal, but could be prevented by dietary phosphate restriction [1] or by adding aluminium salts to their food [2] to bind the phosphate and thereby prevent its absorption. By 1970 the administration of aluminium hydroxide and/or calcium carbonate as phosphate binders in dialysis patients was virtually universal practice.

In 1972, Alfrey was one of the first to report that dialysis patients in his unit in Denver, Colorado, frequently died within a few years due to the development of a severe encephalopathy characterised by seizures, stuttering dysarthria and a distinctive wave and spike pattern on EEG. The term, "dialysis dementia," was coined and Alfrey subsequently demonstrated that all dialysis patients accumulated more aluminium in their tissues than controls with normal renal function and that this accumulation was particularly marked in patients dying of dialysis dementia. He correctly stated that the cause of dialysis dementia was the accumulation of aluminium in the grey matter of the brain [3].

Although aluminium hydroxide phosphate binders were suggested to be the source of this aluminium toxicity, the hypothesis did not explain that while the use of aluminium to bind phosphate was almost universal, the problem of dialysis dementia appeared to be confined, at least initially, to Alfrey's unit. This may have been due to his acute awareness of the toxicity of phosphorus, and more aggressive treatment with aluminium binders, or it may have been due to the aluminium toxicity not coming from binders. An eloquent study by Candy *et al *cast further doubt for some on the theory of the aluminium accumulation being due to binders. This post-mortem study showed that the administration of oral aluminium in a dosage of several grams daily led to the absorption of only microgram quantities of aluminium. The frontal cortex in the brains of dialysis patients exposed to aluminium-based phosphate binders had a mean aluminium content of just 1.2 to 14.1 μg/g dry weight, despite serum levels of aluminium between 5 and 49.9 μg/L [4]. Only half of the patients studied had higher brain aluminium content than control patients. In terms of the elevation of serum levels generated by oral aluminium ingestion and its effect of serum phosphate, one study of 41 patients on haemodialysis revealed that 0.9 grams of aluminium daily reduced the phosphate from 2.10 to 1.48 mmol/L and caused the serum aluminium to rise from 6.8 to 13.8 μg/L (0.25 to 0.51 μmol/L) [5]. In our hospital serum aluminium levels in patients taking oral aluminium are less than 40 μg per litre (< 1.5 μmol/L) in 98% of samples, consistent with reported rates from elsewhere in the world. The direct quantification of absorption of aluminium is difficult in humans due to the lack of stable isotopes able to be measured.

In contrast, evidence emerged that exposure to aluminium in dialysate was far more likely to promote aluminium toxicity than oral exposure. Aluminium crosses the dialysis membrane readily and is 80% plasma protein bound, such that it continues to dialyse into the patient until the plasma aluminium concentration is approximately four times the dialysate concentration [6]. In 1979 a study was published in the UK involving 18 dialysis centres [7] in which the concentration of aluminium in the water used to make dialysate was measured at each centre and correlated with the incidence of dialysis encephalopathy and fracturing osteodystrophy. Those units with the highest concentration of aluminium in the water were also the units with the highest incidence of aluminium toxicity.

The most direct link between aluminium in the water used to prepare dialysis fluid and dialysis dementia came from Eindhoven in Holland [8], Six patients dialysing at one centre in that town developed dialysis dementia. The dialysate was prepared by adding water to the dialysate salts. Part of the water was ordinary tap water while part was obtained from the hospital boiler. The two were mixed to provide an ultimate temperature in the dialysate tank of 40°C. The hospital boiler contained two aluminium anodes as a protection against corrosion. A 3 amp current passed between these anodes and the boiler wall. Over a two year period, the aluminium anodes (which weighed 32 kg) disintegrated completely. They ended up as aluminium hydroxide on the bottom of the hospital boiler. As a result of this, the aluminium content of the dialysis fluid was extremely high. In a second dialysis unit in Eindhoven, dialysate preparation was similar, but there were no aluminium anodes in the boiler and no cases of dialysis dementia occurred.

An outbreak of aluminium toxicity subsequently occurred in Edinburgh in 1978/79 [9] whereby 12 patients developed microcytic anaemia despite normal iron stores. Seven patients developed fracturing osteodystrophy and 1 developed dialysis dementia. The serum aluminium levels in the 12 patients with microcytic anaemia ranged from 7 to 22 μmol/L, whilst the water used to prepare the dialysate had a measured aluminium content which ranged from 0.8 μmol/L to 9.8 μmol/L and was thus identified as the source of aluminium toxicity. A reverse osmosis unit was installed to remove all aluminium from the water. After 15 months of dialysis against aluminium-free dialysate, there was a marked improvement in both serum aluminium levels and microcytic anaemia.

By the 1980s it became clear that the most important cause of epidemic aluminium toxicity was contamination of the water used to make dialysate. Robson *et al *[10] reported improvement in four patients with aluminium-related bone disease following dialysis against aluminium-free dialysate, with stabilisation of a further three cases. No new cases of aluminium-related bone disease occurred after the introduction of reverse osmosis treatment of the water supply, which eliminated aluminium contamination.

There are very limited data to examine the relative contributions of oral and dialysate aluminium to toxicity over time, particularly in the era of more rigorous removal of aluminium from dialysis water. One important study from the late 1990's does shed some light on this, however. Mazzaferro *et al *[11] examined blood and bone aluminium levels over three time periods - 1984 to 1987, 1998 to 1991 and 1992 to 1995, in 105 haemodialysis patients in Italy. In the last time period the aluminium content of the dialysate was controlled by reverse osmosis and was under 10 μg per litre. The average aluminium content in bone halved between the first and last periods and aluminium-related bone disease did not occur after 1992 *in spite of *the continued used of oral aluminium hydroxide to bind phosphate, at doses of 2.6 ± 2.2 g/day. Whilst this paper can not prove the safety of oral aluminium, it is consistent with the theory that aluminium in dialysis water is a stronger risk factor for aluminium accumulation than oral aluminium binders.

While sporadic cases of aluminium toxicity may have been due to the administration of aluminium as a phosphate binder, it is clear that epidemics of aluminium toxicity were due to aluminium in the dialysis fluid. Where toxicity occurs in patients not yet on dialysis, the dialysis fluid obviously cannot be blamed. Such cases have occurred, but they have been limited to children and to CKD patients taking citrate in addition to aluminium.

Children seem to absorb aluminium more readily than adults and there are several reports of children with renal failure developing aluminium toxicity from aluminium-containing phosphate binders prior to commencing dialysis [12]. Infants given aluminium-containing antacids showed significant aluminium absorption compared to controls, as shown by blood and urine aluminium levels [13]. Furthermore, a case series of three infants who were not yet on dialysis, but treated with aluminium developed osteomalacia and accumulation of aluminium in bone on bone biopsy [14]. Plasma aluminium levels in these infants averaged 6.6 μmol/L. This paper, published in the *New UK Journal of Medicine*, was probably one of the most influential in warning that aluminium should therefore be avoided in young children, or at least given with extreme caution.

Citrate was found to increase aluminium absorption from the gut. In the mid-1980's, Bakir *et al *[15] reported the development of dialysis dementia in four patients who had been treated concurrently with oral aluminium and Shohl's solution, a combination of citric acid and sodium citrate. Two of these patients were not yet on dialysis. All four patients had very high serum aluminium levels and all four died. A similar report from elsewhere in the USA [16] detailed a series of eight patients who died within a 6-month period from a syndrome similar to dialysis dementia. They had been receiving aluminium hydroxide concurrently with a citrate solution used to control metabolic acidosis. Post-mortem serum aluminium levels were markedly elevated in two of the patients. Consequently, aluminium levels were measured routinely in that same unit thereafter and the only dialysis patients found to have high levels after that time were those on Shohl's solution in combination with aluminium-containing phosphate binders, or four other patients receiving deferoxamine as therapy for aluminium overload. Three female patients with alterations in mental state after this time had reversal of their symptoms with cessation of the Shohl's solution and aluminium.

In studies in the rat, Froment *et al *[17] showed that the co-administration of citrate and aluminium increased urinary aluminium excretion 50-fold compared with the administration of aluminium alone. The reason for this is that aluminium citrate is much more soluble at physiological pH than either aluminium hydroxide or aluminium chloride. Further studies in humans identified that the high solubility of aluminium citrate enhanced gastrointestinal absorption of aluminium in the presence of citrate, and was responsible for the markedly elevated levels of aluminium and consequent neurological symptomatology seen in patients taking citrate supplements. Alfrey, Froment and others [18] were also able to demonstrate a reduction in aluminium levels after withdrawal of citrate, despite continuation of aluminium binders as the final evidence of this interaction being the cause of the syndrome. Shortly afterwards, citrate ceased to be used as treatment for uremic acidosis and bicarbonate was used instead.

There remain no reported cases of neurological toxicity due to aluminium binders in the absence of concomitant use of citrate in adult pre-dialysis CKD patients in the literature, and importantly, no reports of aluminium toxicity associated with low blood levels of aluminium (<1.5 μmol/L). It is possible therefore, that a threshold of aluminium in the blood such as this level could be chosen and tested prospectively as a safety end-point.

### 2. Contemporary use of aluminium-based binders in the era of greatly improved water quality has not been associated with epidemics of aluminium toxicity

**Figure 1:**
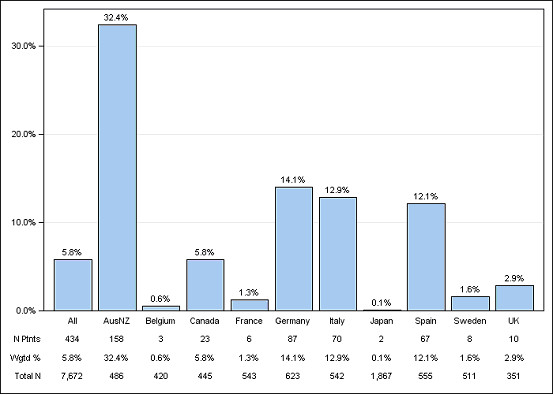
**Aluminium binder use by country in DOPPS 3, 2007**. (source: http://www.dopps.org/annualreport/html/pb_aub_c_mostrec2009.htm)

Aluminium-based phosphate binders have continued to be used not only in Australia but elsewhere in the world, albeit less commonly in Europe and very little in North America. The Dialysis Outcomes and Practice Patterns Survey (DOPPS) reports on aluminium usage, and as recently as the third survey (2007) found substantial usage of aluminium binders in Germany, Italy and Spain, as well as in Australia (Figure 1).

We continue to use aluminium, but principally as a second-line agent after calcium carbonate. At any given time, there would be approximately 200 dialysis patients taking aluminium hydroxide. However, in spite of constant vigilance for clinical and laboratory manifestations of aluminium overload, the last identified case of aluminium toxicity occurred 25 years ago in a patient with dialysis dementia who had serum aluminium levels between 2 and 3 μmol/L and had dialysed at home against a dialysate containing a measured aluminium level of 1 μmol/L. A similar experience had been observed in Edinburgh where the majority of patients who experienced aluminium toxicity had serum levels in excess of 6 μmol/L.

Serum aluminium concentrations are monitored every 3 months in our unit and aluminium hydroxide is discontinued when the serum level is over 1.5 μmol/L. This level is currently encountered in less than 2% of our dialysis population. These findings are in keeping with contemporary data from the US, whereby elevated aluminium levels were identified in 2.1% and 2.5% of large cohorts of hemodialysis patients tested in 2000 and 2003, respectively [19]. That study also noted that the frequency of detection of elevated aluminium levels was actually on the decline, possibly related to better water treatment. A UK study of aluminium testing in dialysis patients queried whether such testing was superfluous, [20] although the dialysis patients in that study were not taking any aluminium-based binders and in that situation aluminium testing of the dialysis water supply only could be argued to be an acceptable minimum safety requirement. On the other hand, given the rare possibility of alternative means of heightened dietary aluminium exposure, such as with inadvertent citrate consumption, continuing to test aluminium levels could be seen to be a safer strategy. An unanswered question is whether an increase in serum aluminium levels over time indicates tissue accumulation of aluminium, and if so, at what serum aluminium level therapy should be withdrawn. This is a question for which there are no published data. Single measurements of serum aluminium do show correlation with aluminium bone disease (ABD) with one study showing a 3-fold higher risk for ABD in those patients in the highest quartile of serum aluminium [21]. However in that study, there was no threshold level of aluminium which discriminated between those patients with ABD and those without. There is however not a single case in the literature of significant aluminium toxicity occurring in the presence of serum aluminium levels of less than 1.5 μmol/L, so it is possible that this level of aluminium could be a starting point for a safety threshold.

In terms of aluminium-related bone disease, this entity is no longer clinically observed at our unit either. Although bone biopsies are not routinely undertaken in all hemodialysis patients, we have, as part of a research project into bone histology in patients converting to alternate nightly haemodialysis, performed bone biopsies in a cohort of 26 patients [22]. The majority of these individuals had normal or increased bone turnover but none had signs of aluminium accumulation, despite the majority having been on long-term aluminium-based binder therapy at some stage prior to the study. The only other contemporary data published on the continued use of aluminium binders in the era of ultra pure water was the aforementioned Spanish study [5] which looked at a cohort of hemodialysis patients continuing to take aluminium-based binders but with monitoring of serum and water aluminium levels from 2005 to 2007. In that investigation, around one in six patients were taking aluminium hydroxide (233 mg tablets) for a mean duration of 17 months, at a mean dose of 3.9 ± 2.3 tablets/day. The serum aluminium levels rose from 6.8 to 13.8 μg/L on average, with no patients exceeding the 40 μg/L safety threshold or developing evidence of toxicity, such as microcytosis or anaemia. This level is similar to the 1.5 μmol/L level discussed previously.

### 3. Aluminium-based binders are effective and cheap

Aluminium remains an effective phosphate binder, and may be safe in an adult dialysis population provided blood levels are carefully and regularly monitored and the agent is withdrawn if these levels rise. Used as a phosphate binder, aluminium will usually reduce serum phosphate by approximately 0.3 mmol/L. A reduction of this amount may equate to a mortality reduction of 10 to 15%, if the data on attributable risk to hyperphosphatemia [23] are accurate. Looking at this another way, failure to use this effective phosphate binding agent could be costing one or two lives per year in the average-sized dialysis unit.

In terms of phosphate targets, the K/DOQI guidelines [24] recommend that predialysis phosphate levels be run at 1.13 to 1.78 mmol/L, even though they readily acknowledge that such targets are difficult to achieve in practice. For example, in the study by Cheng et al [25] of 33 patients, 76% of whom received sevelamer in an average dose of 7.7 grams daily, the mean serum phosphate concentration was 2.3 mmol/L. In our hospital, the mean predialysis serum phosphate level in the haemodialysis population is 1.7 mmol/L. Some of our patients are on haemodiafiltration, and our average dialysis duration is 13.5 hours per week, both of which would be expected to have a beneficial impact on phosphate control compared with centres which only perform short hours haemodialysis. However, despite the use of oral aluminium hydroxide in addition to other agents, we manage to achieve the K/DOQI phosphate target of <1.75 mmol/L in only 55% of our dialysis patients (unpublished data). With the less potent agents such as calcium or sevelamer, we would be unlikely to achieve as good a result without a greater pill burden. This is consistent with the previously mentioned contemporary Spanish study of patients taking aluminium binders, the proportion of patients with a serum phosphate levels <5 mg/dL (1.25 mmol/L) rose from 4.9% to 73.2% when aluminium was used in combination with either sevelamer or calcium [5], reinforcing the effectiveness of adjuvant therapy with aluminium hydroxide in achieving serum phosphate targets.

In contrast, the K/DOQI guidelines caution against the use of aluminium-containing phosphate binders altogether and only recommend their use as a short-term therapy (≤4 weeks) in patients with serum phosphorus levels >7.0 mg/dL (2.26 mmol/L), and for one course only, to be replaced thereafter by other phosphate binders [24]. Moreover, the K/DOQI guidelines recommend that the "total dose of elemental calcium provided by the calcium-based phosphate binders should not exceed 1,500 mg/day (opinion), and the total intake of elemental calcium (including dietary calcium) should not exceed 2,000 mg/day (opinion)." If these guidelines are followed, there is likely to be a substantial shift in clinical practice away from the use of calcium- and aluminium-based phosphate binders to non-calcium-based phosphate binders, such as sevelamer and lanthanum, which are considerably more expensive. Manns et al [26] estimated that compliance with the guidelines will lead to 64% of dialysis patients in the USA (where aluminium is largely not used at all) being prescribed sevelamer, at a staggering additional cost of US$ 781 million per annum. A subsequent economic evaluation of sevelamer in CKD patients considering 4 separate Markov modelling strategies based on data obtained from the DCOR study [27] found that the use of sevelamer was associated with a cost per quality-adjusted life year (QALY) gained that exceeded what would usually be considered good value for money (CAN$105,000 - CAN$278,100). The authors concluded that "this strategy remains economically unattractive, particularly given the uncertainty of clinical benefit in this group." The exact costs and benefits of the use of newer binders remain to be seen, but it may take some years to realise their potential benefits of cost-savings related to lower CKD-MBD morbidity.

In terms of comparative studies of different phosphate binders, there are several worthy of mention. One small randomised controlled trial of aluminium hydroxide versus both calcium carbonate and calcium acetate [28], concluded that "aluminium tended to be the most effective phosphate binder" and also noted that in most patients taking either form of calcium, additional aluminium was necessary to adequately control phosphate. Over the 12 month duration of the study, there was no difference in serum aluminium levels in patients who were taking aluminium compared to those who were not. This probably reflected improvements in water treatment to remove aluminium, such as the use of double reverse-osmosis units, which all but eliminate aluminium from the water used to make dialysate. However, other than serum aluminium levels, no other specific safety criteria for the use of aluminium were analysed (such as bone biopsies, fracture rates or neurological events).

In peritoneal dialysis patients, one small, short-term study of aluminium versus sevelamer [29] found no significant difference between the two interventions in terms of phosphate lowering or side effects, although the phosphate levels were numerically (although not statistically significantly) lower in the patients taking aluminium. Of note, there were only 15 patients in each group of this cross-over study, so the numbers may have been too small to reveal a true difference. Aluminium levels were not measured in this study, despite aluminium hydroxide doses as high as 5.7 g per day being used.

Currently in Australia, the relative costs of aluminium hydroxide versus sevelamer or lanthanum for a typical daily dose are 72¢ per day versus $10.80 or $13.20 per day, respectively. This is for typical doses of 2 tablets taken three times per day with meals for aluminium or sevelamer, or one 750 mg tablet of lanthanum three times daily based on costs at our hospital pharmacy. Over a year this represents costs of $262 per patient for aluminium, and $3942 or $4818 for sevelamer and lanthanum respectively, or a cost differential of between $3680 and $4556 per patient per year. For a dialysis unit with 100 patients this is clearly a very significant expense.

Safety however is obviously a more important consideration than cost. From the DOPPS Studies, it is clear that many thousand dialysis patients have received aluminium as a phosphate binder over the last 15 to 20 years. Despite the strategy not being formally tested in a prospective trial, there is not a single paper in the literature from this period describing significant aluminium toxicity in an adult patient whose aluminium level was regularly monitored and where the drug was discontinued if the plasma level reached 1.5 μmol/L. This strategy could be used as the basis for testing the safety of aluminium binders in a prospective trial.

## Summary

In summary, the historical data which have raised concern about neurological and bone toxicity of aluminium-based phosphate binders may no longer be relevant to contemporary practice. This is due largely to the more rigorous treatment and testing of dialysis water in most haemodialysis units, and also the regular measurement of serum aluminium levels in dialysis patients taking aluminium binders. However, due to the difficulties in measuring aluminium accumulation in humans directly, toxicity of aluminium remains a concern. Serum aluminium levels in dialysis patients receiving aluminium binders are higher than in those not receiving aluminium binders, suggesting some accumulation, the toxicity of which has not been adequately studied.

Epidemics of aluminium toxicity causing neurological toxicity are fortunately no longer seen in contemporary haemodialysis practice, and less severe toxicities such as anemia and aluminum-related bone disease appear to be decreasing with time in centres where it is being looked for. The relative contribution of aluminium binders to aluminium toxicity would appear to be minor based on the available evidence.

Newer phosphate binders, such as sevelamer and lanthanum, are limited by both lack of evidence of benefit in terms of hard clinical end-points (cardiovascular disease, mortality) but also by lack of long-term safety data. Lanthanum accumulation has been well documented in patients taking lanthanum carbonate [30,31], although the long-term effects on patient safety are uncertain. Likewise, such safety data for aluminium binders and even calcium binders are lacking. Indeed, there are no head-to-head trials of different phosphate binders comparing their safety, efficacy (especially with respect to patient-level outcomes) and cost-effectiveness. Contemporary guidelines however, would tend to promote the newer, more costly agents. The onus is on the nephrology community to perform trials to establish high level evidence relating to all aspects of management of hyperphosphatemia including the benefits of lowering serum phosphate per se, as well as questions relating to specific agents and the associated health economic considerations. Moreover large trials which are adequately designed to answer these important questions, should consider all available phosphate binders, including aluminium.

The utility of safety standards of routine water testing for aluminium and monitoring of serum aluminium levels for patients on oral aluminium-based phosphate binders, as well as the avoidance of oral citrate in patients taking aluminium, could reasonably be tested in a prospective, randomised trial, and provides a tantalising opportunity for a potentially significant cost-saving for dialysis patients if the safety of aluminium as a binder could be established.

## Conflict of Interest Statement

David Johnson has served on the consultant advisory boards for Genzyme (manufacturer of sevelamer) and Amgen (manufacturer of cinacalcet). He has received speakers' honoraria, research funding and travel sponsorships from Amgen, as well as speakers' honoraria from Shire (manufacturer of lanthanum). Carmel Hawley has served on advisory boards for Genzyme, Amgen and Shire. She has received research funding and travel sponsorships from Amgen and speakers' honoraria from Shire. David Mudge has received speakers' honoraria from Shire. James Petrie, Scott Campbell, Nicole Isbel, and Carolyn Van Eps declare that they have no conflict of interest.

## Authors' contributions

DWM and JJP conceived and wrote the manuscript. JJP provided the historical references and perspective. DWJ provided editorial input and overall direction. CMH, SBC, NI and CVE helped draft the manuscript and provided additional editorial input. All authors read and approved the final manuscript.

## Pre-publication history

The pre-publication history for this paper can be accessed here:

http://www.biomedcentral.com/1471-2369/12/20/prepub
